# Relationship between Renal Artery Stenosis and Severity of Coronary Artery Disease in Patients with Coronary Atherosclerotic Disease

**Published:** 2012-09-15

**Authors:** Amirfarhang Zandparsa, Mehrdad Habashizadeh, Ehsan Moradi Farsani, Mostafa Jabbari, Razieh Rezaei

**Affiliations:** 1 Department of Cardiology, Tehran University of Medical Sciences, Tehran, IR Iran

**Keywords:** Coronary Artery Disease, Renal Artery Stenosis, Hypertension

## Abstract

**Objective:**

The aim of the present investigation was to explore probable association of renal artery stenosis (RAS) with coronary artery disease (CAD) and the prevalence of renal artery stenosis (RAS) in patients with CAD.

**Patients and methods:**

This study comprised 165 consecutive patients with CAD, including 52.7% males and 47.2% females with respective mean ages of 60.3 ±8.9 and 59.5±10.1. The patients underwent simultaneous coronary and renal angiographies, and the lumen reduction of 50% or more was considered as significant stenosis. Indeed, stenosis of more than 70% of the arterial lumen was regarded as severe.

**Results:**

According to our findings, the prevalence of renal artery stenosis in our hypertensive and normotensive patients were 46.2% and 19.5% respectively (p=0.002). Renal artery angiography revealed that 64 (38.8%) of the patients had simultaneous renal artery stenosis. RAS is more common in females than males (p=0.031). Multivariate analysis revealed that among all examined factors, hypertension and serum creatinine were associated with RAS. There was no correlations found between gensini score and RAS (p=0.63).

**Conclusion:**

We found a relatively high prevalence of RAS including 46.2% in hypertensive and 19.5% in normotensive patients in our patients with CAD.

## Introduction

Atherosclerosis is a diffuse process characterized by functional and morphologic changes of the arterial wall including endothelial dysfunction, increased wall thickness and progressive plaque formation (1,2). Although the disease begins early in life it has no symptoms for a long period of time. It usually affects main arteries of body including the coronary, carotid, and renal arteries.

The initial clinical presentation of coronary artery disease (CAD) may be abrupt due to plaque rupture with varying amounts of superimposed thrombus, vasoconstriction, and distal embolization, leading to acute coronary events, such as angina pectoris (AP) or myocardial infarction (MI) (3-5).

As mentioned above, atherosclerosis can occurs in renal arteries leading to renal artery stenosis which is an important cause of secondary hypertension and also a main reason for renal failure in elderly. As investigated, 60-97% of renal artery stenosis in different areas are due to atherosclerosis (6,7) and 10-20% of all reported end stage renal diseases have a background of renal ischemia (7,8).

Angiography is the gold standard method for evaluation of stenosis in arteries in which it is used widely for detecting coronary artery obstruction (1). It is shown that most of the renal artery stenosis is clinically silent, and according to large scale studies about 15% of patients with CAD have concurrent renal artery stenosis, thus it is suggested by some authors that CAD may be a strong predictor for atherosclerotic renal artery stenosis (9-11). Considering the importance of these indolent lesions, the present study attempted to explore the prevalence of renal artery stenosis and its probable association with CAD.

## Patients and Methods

Between September 2010 and May 2011, 165 consecutive patients with CAD admitted to our department for coronary angiography were enrolled in this study. All the patients were selected and underwent coronary angiography according to American Heart Association and American College of Cardiology (AHA/ACC) guidelines. Renal angiography was also performed in all patients and during one session using a Siemens highcore system (Siemens company, Germany). All coronary angiographic examinations were performed by the Seldinger technique through femoral artery access. Angiographies were carried out in several views that best displayed the lesion and enabled stenosis grade evaluation. Intra-arterial systolic and diastolic pressures of the ascending aorta were measured during cardiac catheterization. In patients with creatinine level 1.5 mg/dl or less and significant coronary artery disease , abdominal aortography was conducted using a pigtail catheter with a pump injector. Whenever difficult to evaluate the degree of stenosis , selective renal arteriography was performed with a right 6 or 7 French Judkins catheter in the anterior–posterior, and when necessary in oblique projection. Lumen reduction of 50% and more was considered as significant stenosis. Indeed, stenosis more than 70% of the arterial lumen was considered as severe stenosis. All angiograms were analyzed by an experienced interventional cardiologist and together with another analyst who reviewed the angiograms reached a consensus. Patients were classified as having single vessel disease (1VD), two vessles disease (2VD) and three vessels disease (3VD) if they had a significant stenosis in 1, 2, and 3 major epicardial coronary vessels . Left main lesions counted as 2VD were considered present when luminal diameter was reduced by 50%.

Fasting blood sugar (FBS), serum total cholesterol, low-density lipoprotein cholesterol (LDL), high-density lipoprotein cholesterol (HDL), triglycerides (TG) and creatinine were measured in all patients (BT 3000, Biotecnica Instruments, Rome, Italy). Hypercholesterolemia denoted total serum cholesterol more than 240 (mg/dl), and hypertriglyceridemia indicated triglycerides more than 200 (mg/dl). High LDL and low HDL were defined as more than 160 (mg/dl), and less than 35 (mg/dl) respectively. Patients with blood pressures higher than 140/90 mmHg or documented history of hypertension or antihypertensive therapy were considered as hypertensive. Serum creatinine level ≤1.4 mg/dl considered as normal value and patients with creatinine level >1.5 excluded from this study as well as patients who had undergone previous renal or coronary revascularization.

Gensini’s score was also calculated for each patient as previously described (12). Patients with FBS greater than 126 mg/dl or documented history of medication for diabetes mellitus (DM) were considered as diabetic. Demographic data including age and related family history of CAD were also collected using a standard questionnaire. An established positive family history (FH) for CAD was based on a known history of CAD in a first-degree relative male or female aged less than 55 and 65 years. The study was approved by Tehran University of Medical Sciences Ethics committee, and written informed consent was obtained from all patients willing to participate in the study.

## Statistical analysis

Statistical analysis was performed using SPSS version 16.0.1 (SPSS Inc., Chicago, IL, U.S.A.). Normality of data was evaluated with the Kolmogorov–Smirnov test. The results were expressed as mean ± SD for parametric and mean ± SEM for nonparametric data. The statistical differences between proportions were determined by χ2 analysis. Numerical data were evaluated using analysis of variance, followed by Tukey’s post hoc test. P value less than 0.05 was considered as significant. Pearson and Spearman correlation tests were used for parametric and nonparametric numeric data analysis respectively.

## Results

Among 165 patients 52.7% were males and 47.2% females with respective mean ages of 60.3±8.9 and 59.5±10.1 years, and without any statistically significant difference between them. Of the patients under study,71 (43%) had diabetes mellitus (DM), 119 (72.1%) were hypertensive (HTN), 129 (78.2%) had hyperlipidemia (HLP), 63(38.2%) were cigarette smokers and 38 (23%) had family history of CAD. Patients’ creatinine level was 1.15±0.2. Of 165 (6.1%) patients,10(6.1%) had creatinine levels higher than normal range. Table 1 shows detailed demographic data of all patients.

**Table 1:** Patients’ Clinical and Demographic Characteristics.

**Age (mean ±SD a)**		59.9 ±8.9
**Gender (male/female)**		87 (52.7%) / 78 (47.2%)
**Diabetes mellitus**		71(43%)
**Hypertension**		119 (72.1%)
**Hyperlipidemia**		129 (78.2%)
**Smoking**		63 (38.2%)
**Family history of CAD a**		38 (23%)
**Serum creatinine mg/dl, (mean ±SD)**		1.15 ±0.19
**Systolic blood pressure (mmHg) (mean ±SD)**		151.7 ±27
**Diastolic blood pressure (mmHg) (mean ±SD)**		82 ±12.5
**Renal artery stenosis**		64 (38.8%)
**Involved coronary arteries**	**One vessel**	50 (30.3%)
	**Two vessels**	33 (20%)
	**Three vessels**	82 (49.7%)
**Ejection fraction**		54.2 ±9.9
**Gensini's score (mean ±SEM a)**		36.15 ±2.6

^a^Abbreviations: SD, standard deviation; CAD, coronary artery disease; SEM, standard error of the mean

## Angiography

Coronary angiography revealed that 50 patients (30.3%) had one vessel involved by atherosclerosis, 33 (20%) had 2 vessels, and 82 (49.7%) had three vessels disease. Seven patients(4.2%) had more than 50% stenosis in left main coronary artery that categorized as 2 vessels in absence of significant right coronary stenosis and 3 vessels with significant right coronary stenosis or left side dominancy. Renal artery angiography revealed that 64 (38.8%) of the patients had simultaneous renal artery stenosis which in 26 patients it was in the right renal artery, 26 in the left and 12 (7.3%) in both renal arteries. RAS was present in 64 patients, which was severe in 26 (40.6%). Results showed that RAS is more common in females than males (P=0.031). There was no relationship found between the number of stenotic coronary arteries and RAS severity (P>0.05). Also no correlations were observed between severity of coronary artery disease (gensini’s score) and renal artery stenosis (P=0.63)

The means systolic and diastolic blood pressures of patients which measured during angiography were 151.7±27.5 and 82±12.5 respectively. There was no significant difference between the mean age of patients with or without RAS (P=0.9).However, the mean age of patients with HTN was significantly higher than normotensive patients (P<0.001), and the stratifying data for RAS revealed the same result, showing that the mean age of patients with HTN was significantly higher than normotensive patients in both RAS and non-RAS patients (P=0.001).

Also the prevalence of RAS in hypertensive patients was more than normotensive patients (P=0.002). Indeed, patients with RAS had higher systolic and diastolic blood pressures and serum creatinine level with respective P values of 0.009, 0.006 and <0.001.

As described above, we showed a total prevalence of 38.8% RAS in our patients. The prevalence of RAS in our hypertensive and normotensive patients were 46.2% and 19.5% respectively. The patients’ ejection fraction (EF) was 54.2% ±9.9. Multivariate analysis revealed that among all factors examined, hypertension and serum creatinine were associated with RAS, where in patients with CAD, higher blood pressure and higher serum creatinine levels are predisposing factors(p=0.009 and p=0.03 , respectively). There was no association between RAS and DM, HLP, smoking or family history with respective P values of 0.23, 0.15 , 0.13 and 0.87.

The mean value of gensini score was 36.1±2.6. Analysis showed that serum creatinine level and cardiac EF was correlated with gensini scores (P=0.002, correlation coefficient=0.24 and P<0.001, correlation coefficient= -0.4, respectively). However, no correlations were found between gensini’s score and RAS percentage, RAS, systolic or diastolic blood pressure ( Table 2).

**Table 2:** Patients’ Clinical and Angiographic Characteristics Data.

Clinical and angiographic characteristic		RAS a(64)	Non-RAS(101)	P value
**Age**		60.4±9.6	59.5±9.5	0.9
**Gender (female/male)**		37/27 (57.8%/42.1%)	41/60 (40.5%/59.4%)	0.031
**Diabetes mellitus**		25 (39%)	46 (45.5%)	0.23
**Hypertension**		55 (85.9%)	64 (63.3%)	0.002
**Hyperlipidemia**		49 (76.5%)	80 (79.2%)	0.15
**Smoking**		29 (45.3%)	34 (33.6%)	0.13
**Family history of CAD a**		17 (26.5%)	21(20.7%)	0.87
**Systolic blood pressure**		159.1±30.8	147 ±24	0.009
**Diastolic blood pressure**		84.7±14.7	78.7±10.3	0.006
**Serum creatinine level**		1.22±0.16	1.1±0.2	<0.001
**Gensini score**		40.2±4.4	33.5±3.2	0.63
**Involved coronary arteries**	**One vessel**	18 (28.1%)	32 (31.6%)	0.12
	**Two vessels**	15 (23.4%)	18 (17.8%)	0.43
	**Three vessels**	31(48.4%)	51(50.4%)	0.66
**Ejection fraction**		55.5±10	53.5±10	0.12

^a^Abbreviations: RAS, renal artery stenosis; CAD, coronary artery disease

## Discussion

In the current study we explored the prevalence of RAS in patients with CAD and evaluated the relationships between RAS and CAD. Recent studies indicated that RAS was not a rare finding in patients with coronary artery disease and our results showed that about 39% of our patients with CAD had RAS besides CAD. Considering HTN, our results showed that the prevalence of RAS in patients with no history of HTN was about 19.5%. This finding was compatible with other studies reporting such prevalence rate as 15.7% to 42%. Some of these studies have used non-invasive methods to determine the prevalence of RAS and this is one of the reasons for high RAS prevalence found in their patients. However, some other studies showed lower prevalence of RAS (7,9,13,14). Our Results showed that RAS was more common in females than males, which is compatible with another study (15). However, additional investigation indicated that male gender was an independent pedictor of RAS (16). Our results revealed that the means of systolic and diastolic blood pressures and serum creatinine level were higher in RAS than in those without RAS. Also the prevalence of hypertension was higher in RAS patients. Some studies demonstrated that RAS was usually indolent and most of the patients with RAS were not hypertensive. Although in our study the patients with RAS had significantly higher blood pressures, nevertheless we also found a high prevalence of RAS among normotensive patients.

Multivariate analysis revealed that there was strong association of RAS with hypertension and serum creatinine level. A trend towards a significant relationship between hypertension and the presence of RAS has previously been established. Hypertension was shown to be a risk factor for RAS as well as a possible clinical manifestation of the activated renin angiotensin system secondary to RAS (13,17). High serum creatinine level is an indicator of renal insufficiency. Previous studies have shown the association between RAS and renal insufficiency based on creatinine clearance (18), which is consistent with our findings. As renovascular disease can lead to ischemic nephropathy or end stage renal disease, high prevalence of RAS can cause significant mortality and morbidity (19).

Our study showed no association between the number of coronary involved vessels and the severity of the RAS but there are some studies showing significant association between them (13). Association between age and RAS was not seen in our study which was shown elsewhere (13,20). Some studies have introduced CAD as a strong predictor for RAS (1). In the current study, although we found a high prevalence of RAS in CAD patients regardless of hypertension, no relationship was found between gensini score and RAS. Gensini score is previously described as a determinant of coronary artery disease severity (12). Some studies showed an association between the number of cardiac atherosclerotic vessels and RAS (21). In this connection, the presence of more than 2 significant coronary lesions is recognized as an independent predictor of RAS (20).

Our data revealed no association between RAS and DM, HLP, family history of CAD or smoking. These findings are in line with other studies (15,22).

The limitation of our study was that it was performed on a group of patients with confirmed CAD, thus the results obtained cannot be extrapolated to other patients with peripheral vascular atherosclerosis without any evidence of CAD.

We found a relatively high prevalence of RAS, 46.2% in hypertensive and 19.5% in normotensive patients, in our patients with CAD. This finding underlines the importance of further awareness of renal function in patients with CAD. More studies are suggested to determine the relationship between CAD, RAS and HTN, in order to avoid RAS mortality and morbidities among CAD patients. Concurrent renal and coronary angiographies might be of benefit to those with history of hypertension and higher creatinine levels and the patients with increased intra-arterial systolic and diastolic pressures detected during the procedure.
